# Supplementary material for: Integrative Taxonomy of the Spinous Assassin Bug Genus Sclomina (Heteroptera: Reduviidae: Harpactorinae) Reveals Three Cryptic Species Based on DNA Barcoding and Morphological Evidence
Source: Insects. 2021 Mar 16;12(3):251. doi: 10.3390/insects12030251 (PMC8001484; doi:10.3390/insects12030251)
Supplement: Supplementary file 1 [file Figure S1. Bayesian phylogenetic tree of cytochrome c oxidase subunit I (COI) sequence for 307 terminals .pdf › Figure S1. Bayesian phylogenetic tree of cytochrome c oxidase subunit I (COI) sequence for 307 terminals .pdf]

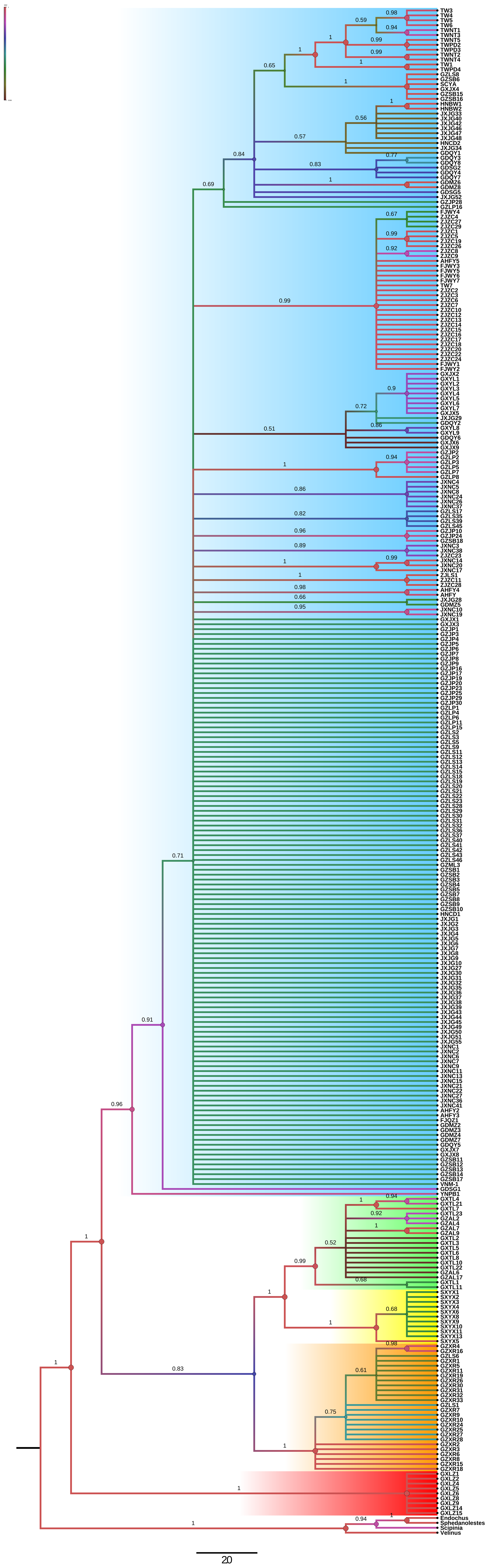

TW3
TW4
TW5
TW6
TWNT1
TWNT3
TWNT5
TWPD2
TWPD3
TWNT2
TWNT4
TW1
TWPD4
GZLS8
GZSB6
SCYA
GXJX4
GZSB15
GZSB16
HNBW1
HNBW2
JXJG33
JXJG40
JXJG42
JXJG46
JXJG47
JXJG48
HNCD2
JXJG34
GDQY1
GDQY3
GDQY8
GDSG2
GDQY4
GDQY7
GDMZ6
GDMZ8
GDSG5
JXJG52
GZJP28
GZLP16
FJWY4
ZJZC4
ZJZC27
ZJZC29
ZJZC1
ZJZC5
ZJZC19
ZJZC26
ZJZC8
ZJZC9
AHFY5
FJWY3
FJWY5
FJWY6
FJWY7
TW7
ZJZC2
ZJZC3
ZJZC6
ZJZC7
ZJZC10
ZJZC12
ZJZC13
ZJZC14
ZJZC15
ZJZC16
ZJZC17
ZJZC18
ZJZC20
ZJZC22
ZJZC24
FJWY1
FJWY2
GXJX2
GXYL1
GXYL2
GXYL3
GXYL4
GXYL5
GXYL6
GXYL7
GXJX5
JXJG29
GDQY2
GXYL8
GXYL9
GDQY6
GXJX6
GXJX9
GZJP2
GZLP2
GZLP3
GZLP5
GZLP7
GZLP8
JXNC4
JXNC5
JXNC8
JXNC24
JXNC26
JXNC37
GZLS17
GZLS35
GZLS39
GZLS45
GZJP10
GZJP24
GZSB18
JXNC3
JXNC38
ZJZC23
JXNC14
JXNC20
JXNC17
ZJLS1
ZJZC11
ZJZC28
AHFY4
AHFY
JXJG28
GDMZ5
JXNC10
JXNC19
GXJX1
GXJX3
GZJP1
GZJP3
GZJP4
GZJP5
GZJP6
GZJP7
GZJP8
GZJP9
GZJP16
GZJP17
GZJP19
GZJP20
GZJP23
GZJP25
GZJP29
GZJP30
GZLP1
GZLP4
GZLP6
GZLP11
GZLP15
GZLS2
GZLS3
GZLS5
GZLS9
GZLS11
GZLS12
GZLS13
GZLS14
GZLS15
GZLS18
GZLS19
GZLS20
GZLS21
GZLS22
GZLS23
GZLS28
GZLS29
GZLS30
GZLS31
GZLS32
GZLS36
GZLS37
GZLS40
GZLS41
GZLS42
GZLS43
GZLS46
GZML3
GZSB1
GZSB2
GZSB3
GZSB4
GZSB5
GZSB7
GZSB8
GZSB9
GZSB10
HNCD1
JXJG1
JXJG2
JXJG3
JXJG4
JXJG5
JXJG6
JXJG7
JXJG8
JXJG9
JXJG10
JXJG27
JXJG30
JXJG31
JXJG32
JXJG35
JXJG36
JXJG37
JXJG38
JXJG39
JXJG43
JXJG44
JXJG45
JXJG49
JXJG50
JXJG51
JXJG55
JXNC1
JXNC2
JXNC6
JXNC7
JXNC9
JXNC11
JXNC13
JXNC15
JXNC21
JXNC22
JXNC27
JXNC36
JXNC41
AHFY2
AHFY3
FJQZ1
GDMZ2
GDMZ3
GDMZ4
GDMZ7
GDQY5
GXJX7
GXJX8
GZSB11
GZSB12
GZSB13
GZSB14
GZSB17
VNM-1
GDSG1
YNPB1
GXTL4
GXTL21
GXTL7
GXTL23
GZAL2
GZAL4
GZAL7
GZAL9
GXTL2
GXTL3
GXTL5
GXTL6
GXTL8
GXTL10
GXTL22
GZAL6
GZAL17
GXTL1
GXTL11
SXYX1
SXYX2
SXYX3
SXYX4
SXYX6
SXYX8
SXYX9
SXYX10
SXYX11
SXYX13
SXYX5
GZXR4
GZXR16
GZLS6
GZXR1
GZXR5
GZXR11
GZXR19
GZXR26
GZXR30
GZXR31
GZXR32
GZXR33
GZLS1
GZXR7
GZXR9
GZXR10
GZXR24
GZXR25
GZXR27
GZXR28
GZXR2
GZXR3
GZXR6
GZXR8
GZXR15
GZXR18
GXLZ1
GXLZ2
GXLZ4
GXLZ5
GXLZ6
GXLZ8
GXLZ9
GXLZ14
GXLZ15
Endochus
Sphedanolestes
Scipinia
Velinus
20
