# Supplementary material for: Integrative Taxonomy of the Spinous Assassin Bug Genus Sclomina (Heteroptera: Reduviidae: Harpactorinae) Reveals Three Cryptic Species Based on DNA Barcoding and Morphological Evidence
Source: Insects. 2021 Mar 16;12(3):251. doi: 10.3390/insects12030251 (PMC8001484; doi:10.3390/insects12030251)
Supplement: Supplementary file 1 [file insects-12-00251-s001.zip › Supporting information.pdf]

Supporting information

Integrative Taxonomy of the Spinous Assassin Bug Genus *Sclomina* (Heteroptera: Reduviidae: Harpactorinae) Reveals Three Cryptic Species Based on DNA Barcoding and Morphological Evidence

Ping Zhao[1,3], Zhenyong Du[2], Qian Zhao[2], Donghai Li[3], Xiaolan Shao[3], Hu Li[2*], and Wanzhi Cai[2*]

[1] Key Laboratory of Environment Change and Resources Use in Beibu Gulf (Ministry of Education) and Guangxi Key Laboratory of Earth Surface Processes and Intelligent Simulation, Nanning Normal University, Nanning, Guangxi 530001, China

[2] Department of Entomology and MOA Key Lab of Pest Monitoring and Green Management, College of Plant Protection, China Agricultural University, Beijing 100193, China

[3] Department of Plant Protection, Kaili University, Kaili, Guizhou 556000, China

* Correspondence: caiwz@cau.edu.cn; tigerleecau@hotmail.com.

Figure S1. Bayesian phylogenetic tree of cytochrome *c* oxidase subunit I (*COI*) sequence for 307 terminals of *Sclomina* (Figure S1 is uploaded separately)

Table S1. GenBank Accession number under every sample individual code (as following)

Table S2. Pairwise genetic divergence (Kimura two-parameter) within and between species of the genus *Sclomina* using cytochrome *c* oxidase subunit I gene sequence (as following)

Table S3. Pairwise genetic divergence (Kimura two-parameter) between 307 individuals of five species of the genus *Sclomina* using cytochrome *c* oxidase subunit I gene sequence (Table S3 is uploaded separately)

Table S4. Pairwise genetic divergence (Kimura two-parameter) within and between 30 sampling groups of the genus *Sclomina* using cytochrome *c* oxidase subunit I gene sequence (Table S4 is uploaded separately)

Table S5. Life history of *Sclomina erinacea* Stål, 1861 (as following)

Table S6. Development time of *Sclomina erinacea* Stål, 1861(26 ℃ ± 2 ℃) (as following)

File S1. Expanded description of *Sclomina erinacea* Stål, 1861 and *Sclomina guangxiensis* Ren, 2001 (as following)

Alignment S1. Alignment of *COI* dataset (Alignment S1 is uploaded separately)

Table S1. GenBank Accession number under every sample individual code

No.	Sample code	GenBank Accession number under every sample individual code
1	AHFY	AHFY (MW387904), AHFY2 (MW387905), AHFY3 (MW387906), AHFY4 (MW387643), AHFY5 (MW387644)
2	FJWY	FJWY1 (MW387908), FJWY2 (MW387909), FJWY3 (MW387645), FJWY4 (MW387646), FJWY5 (MW387647), JWY6 (MW387648), FJWY7 (MW387649)
3	FJQZ	FJQZ1 (MW387907)
4	GDMZ	GDMZ2 (MW387910), GDMZ3 (MW387911), GDMZ4 (MW387912), GDMZ5 (MW387913), GDMZ6 (MW387914), GDMZ7 (MW387915), GDMZ8 (MW387916)
5	GDQY	GDQY1 (MW387917), GDQY2 (MW387918), GDQY3 (MW387919), GDQY4 (MW387920), GDQY5 (MW387921), GDQY6 (MW387922), GDQY7 (MW387923), GDQY8 (MW387924)
6	GDSG	GDSG1 (MW387650), GDSG2 (MW387651), GDSG5 (MW387652)
7	GXJX	GXJX1 (MW387653), GXJX2 (MW387654), GXJX3 (MW387655), GXJX4 (MW387925), GXJX5 (MW387926), GXJX6 (MW387927), GXJX7 (MW387928), GXJX8 (MW387929), GXJX9 (MW387930)
8	GXLZ	GXLZ1 (MW387656), GXLZ2 (MW387657), GXLZ4 (MW387658), GXLZ5 (MW387659), GXLZ6 (MW387660), GXLZ8 (MW387661), GXLZ9 (MW387662), GXLZ14 (MW387663), GXLZ15 (MW387664)
9	XTL	GXTL1 (MW387665), GXTL2 (MW387666), GXTL3 (MW387667), GXTL4 (MW387668), GXTL5 (MW387669), GXTL6 (MW387670), GXTL7 (MW387671), GXTL8 (MW387672), GXTL10 (MW387673), GXTL11 (MW387674), GXTL21 (MW387675), GXTL22 (MW387676), GXTL23 (MW387677)
10	GXYL	GXYL1 (MW387678), GXYL2 (MW387679), GXYL3 (MW387680), GXYL4 (MW387681), GXYL5 (MW387682), GXYL6 (MW387683), GXYL7 (MW387684), GXYL8 (MW387685), GXYL9 (MW387686)
11	GZAL	GZAL2 (MW387687), GZAL4 (MW387688), GZAL6(MW387689), GZAL7(MW387690), GZAL9 (MW387691), GZAL17 (MW387692)
12	GZJP	GZJP1 (MW387693), GZJP2 (MW387694), GZJP3 (MW387695), GZJP4 (MW387696), GZJP5 (MW387697), GZJP6 (MW387698), GZJP7 (MW387699), GZJP8 (MW387700), GZJP9 (MW387701), GZJP10 (MW387702), GZJP16 (MW387703), GZJP17 (MW387704), GZJP19 (MW387705), GZJP20 (MW387706), GZJP23 (MW387707), GZJP24 (MW387708), GZJP25 (MW387709), GZJP28 (MW387710), GZJP29 (MW387711), GZJP30 (MW387712)
13	GZLP	GZLP1 (MW387713), GZLP2 (MW387714), GZLP3 (MW387715), GZLP4 (MW387716), GZLP5 (MW387717), GZLP6 (MW387718), GZLP7 (MW387719), GZLP8 (MW387720), GZLP11 (MW387721), GZLP15 (MW387722), GZLP16 (MW387723)
14	GZLS	GZLS1 (MW387724), GZLS2 (MW387725), GZLS3 (MW387726), GZLS5 (MW387727), GZLS6 (MW387728), GZLS8 (MW387729), GZLS9 (MW387730), GZLS11 (MW387731), GZLS12 (MW387732), GZLS13 (MW387733), GZLS14 (MW387734), GZLS15 (MW387735), GZLS17 (MW387736), GZLS18 (MW387737), GZLS19 (MW387738), GZLS20 (MW387739), GZLS21 (MW387740), GZLS22 (MW387741), GZLS23 (MW387742), GZLS28 (MW387743), GZLS29 (MW387744), GZLS30 (MW387745), GZLS31 (MW387746), GZLS32 (MW387747), GZLS35 (MW387748), GZLS36 (MW387749), GZLS37 (MW387750), GZLS39 (MW387751), GZLS40 (MW387752), GZLS41 (MW387753), GZLS42 (MW387754), GZLS43 (MW387755), GZLS45 (MW387756), GZLS46 (MW387757)
15	GZML	GZML3 (MW387758)
16		GZSB1 (MW387759), GZSB2 (MW387760), GZSB3 (MW387761), GZSB4 (MW387762), GZSB5 (MW387763), GZSB6 (MW387764), GZSB7 (MW387765), GZSB8 (MW387766), GZSB9 (MW387767), GZSB10 (MW387768), GZSB11 (MW387931), GZSB12 (MW387932), GZSB13 (MW387933), GZSB14 (MW387934), GZSB15 (MW387935), GZSB16 (MW387936), GZSB17 (MW387937), GZSB18 (MW387938)
17	GZXR	GZXR1 (MW387769), GZXR2 (MW387770), GZXR3 (MW387771), GZXR4 (MW387772), GZXR5 (MW387773), GZXR6 (MW387774), GZXR7 (MW387775), GZXR8 (MW387776), GZXR9 (MW387777), GZXR10 (MW387778), GZXR11 (MW387779), GZXR15 (MW387780), GZXR16 (MW387781), GZXR18 (MW387782), GZXR19

		(MW387783), GZXR24 (MW387784), GZXR25 (MW387785), GZXR26 (MW387786), GZXR27 (MW387787), GZXR28 (MW387788), GZXR30 (MW387789), GZXR31 (MW387790), GZXR32 (MW387791), GZXR33 (MW387792)
18	HNBW	HNBW1 (MW387939), HNBW2 (MW387940)
19	HNCD	HNCD1 (MW387793), HNCD2 (MW387794)
20	JXJG	JXJG1 (MW387795), JXJG2 (MW387796), JXJG3 (MW387797), JXJG4 (MW387798), JXJG5 (MW387799), JXJG6 (MW387800), JXJG7 (MW387801), JXJG8 (MW387802), JXJG9 (MW387803), JXJG10 (MW387804), JXJG27 (MW387805), JXJG28 (MW387806), JXJG29 (MW387807), JXJG30 (MW387808), JXJG31 (MW387809), JXJG32 (MW387810), JXJG33 (MW387811), JXJG34 (MW387812), JXJG35 (MW387813), JXJG36 (MW387814), JXJG37 (MW387815), JXJG38 (MW387816), JXJG39 (MW387817), JXJG40 (MW387818), JXJG42 (MW387819), JXJG43 (MW387820), JXJG44 (MW387821), JXJG45 (MW387822), JXJG46 (MW387823), JXJG47 (MW387824), JXJG48 (MW387825), JXJG49 (MW387826), JXJG50 MW387827), JXJG51 (MW387828), JXJG52 (MW387829), JXJG55 (MW387830)
21	JXNC	JXNC1 (MW387831), JXNC2 (MW387832), JXNC3 (MW387833), JXNC4 (MW387834), JXNC5 (MW387835),JXNC6 (MW387836), JXNC7 (MW387837), JXNC8 (MW387838), JXNC9 (MW387839), JXNC10 (MW387840), JXNC11 (MW387841), JXNC13 (MW387842), JXNC14 (MW387843), JXNC15 (MW387844), JXNC17 (MW387845), JXNC19 (MW387846), JXNC20 (MW387847), JXNC21 (MW387848), JXNC22 (MW387849), JXNC24 (MW387850), JXNC26 (MW387851), JXNC27 (MW387852), JXNC36 (MW387853), JXNC37 (MW387854), JXNC38 (MW387855), JXNC41 (MW387856)
22	SCYA	SCYA (MW387857)
23	SXYX	SXYX1 (MW387858), SXYX2 (MW387859), SXYX3 (MW387860), SXYX4 (MW387861), SXYX5 (MW387941), SXYX6 (MW387942), SXYX8 (MW387943), SXYX9 (MW387944), SXYX10 (MW387945), SXYX11 (MW387946), SXYX13 (MW387947)
24	TW	TW1 (MW387862), TW3 (MW387863), TW4 (MW387864), TW5 (MW387865), TW6 (MW387866), TW7 (MW387867)
25	YWNT	TWNT1 (MW387868), TWNT2 (MW387869), TWNT3 (MW387870), TWNT4 (MW387871), TWNT5 (MW387872)
26	TWPD	TWPD2 (MW387873), TWPD3 (MW387874), TWPD4 (MW387875)
27	VNM	VNM-1 (MW387948)
28	YNPB	YNPB1 (MW387949)
29	ZJLS	ZJLS1 (MW387876)
30	ZJZC	ZJZC1 (MW387877), ZJZC2 (MW387878), ZJZC3 (MW387879), ZJZC4 (MW387880), ZJZC5 (MW387881), ZJZC6 (MW387882), ZJZC7 (MW387883), ZJZC8 (MW387884), ZJZC9 (MW387885), ZJZC10 (MW387886), ZJZC11 (MW387887), ZJZC12 (MW387888), ZJZC13 (MW387889), ZJZC14 (MW387890), ZJZC15 (MW387891), ZJZC16 (MW387892), ZJZC17 (MW387893), ZJZC18 (MW387894), ZJZC19 (MW387895), ZJZC20 (MW387896), ZJZC22 (MW387897), ZJZC23 (MW387898), ZJZC24 (MW387899), ZJZC26 (MW387900), ZJZC27 (MW387901), ZJZC28 (MW387902), ZJZC29 (MW387903)

Table S2. Pairwise genetic divergence (Kimura two-parameter) within and between species of the genus *Sclomina* using cytochrome *c* oxidase subunit I gene sequence

	**Distance within species**	**Distance between species**				
**Species**		**SE**	**SG**	**SP**	**SX**	**SPA**
**SE**	0.0097					
**SG**	0	0.0915				
**SP**	0.0096	0.0659	0.0924			
**SX**	0.0022	0.0618	0.0975	0.0562		
**SPA**	0.0003	0.0618	0.1053	0.0557	0.0849	

†SE, *Sclomina erinacea* Stål, 1861; SG, *Sclomina guangxiensis* Ren, 2001; SP, ***Sclomina pallens*** **sp. nov.**; SX, ***Sclomina xingrensis*** **sp. nov.**; SPA, ***Sclomina parva*** **sp. nov.**

Table S5. Life history of *Sclomina erinacea* Stål, 1861

	January			February			March			April			May			June			July			August			September			October			November			December		
	A	B	C	A	B	C	A	B	C	A	B	C	A	B	C	A	B	C	A	B	C	A	B	C	A	B	C	A	B	C	A	B	C	A	B	C
Egg											0	0	0	0	0	0																				
1st instar nymph												1	1	1	1	1	1																			
2nd instar nymph													2	2	2	2	2																			
3rd instar nymph														3	3	3	3	3																		
4th instar nymph															4	4	4	4	4	4	4															
5th instar nymph																		5	5	5	5	5	5	5	5	5	5	5								
Adult	(+)	(+)	(+)	(+)	(+)	(+)	(+)	(+)	(+)	+	+	+	+	+	+	+	+					+	+	+	+	+	+	+	+	+	(+)	(+)	(+)	(+)	(+)	(+)

†A: the first 10 days of month; B: the middle 10 days of month; C: the last 10 days of month. ‡ "0": egg; "1" : 1st instar nymth; "2" : 2nd instar nymth; "3" : 3rd instar nymth; "4": 4th instar of nymth; "5": 5th instar of nymth. § "(+)": overwinter adult; "+": adult.

Table S6. Development time of *Sclomina erinacea* Stål, 1861(26 ℃ ± 2 ℃)

**Stages**	**Egg**	**1st instar**	**2nd instar**	**3rd instar**	**4th instar**	**5th instar**
The shortest period (d)	15	2	7	17	32	37
The longest period (d)	19	3	16	21	36	49
Average period (d)	16.93±1.69	2.56±0.53	13.06±3.44	18.6±1.64	34.08±1.56	42.33±3.98

File S1. Expanded description of *Sclomina erinacea* Stål, 1861 and *Sclomina guangxiensis* Ren, 2001

***Sclomina erinacea* Stål, 1861**

(Figures 2a–o; 3a–o; 5; 6a–c; and 7)

Sclomina erinacea Stål, 1861: 137; Hsiao, & Ren, 1981: 490; Maldonado-Capriles, 1990: 294; Putshkov, & Putshkov, 1996: 254.

Diagnosis: *S. erinacea* is the type species of the genus *Sclomina. S. erinacea* resembles *S. guangxiensis* in body shape and size. In the diagnosis section of *S. guangxiensis* in the following, we discuss the main distinguishing characteristics of the two species.

Redescription: Colouration: Body yellowish-brown to brown, with black markings (Figures 7a–c; and 6a, b). Longitudinal strips on the lateral side of head and posterior lobe of head, propleural episternum, markings of meso- and meta-pleuron of thorax, markings of transverse constriction of pronotum, middle part of scutellum, middle part of corium, markings on lateral side of abdomen, inner side of femur, basal part of first antennal segment black; longitudinal strips on fore femur, obscure annular markings of mid and hind femora, oblique strip of basal part of tibia, tarsus, middle part of each connexival segment brown to blackish-brown; first antennal segment reddish-brown, middle part with two pale annuli, second segment pale brown, sub-apical part pale and apical part black, third segment brown, sub-basal part pale and basal part black, fourth segment yellowish-brown; posterior 2/3 of clavus and membrane semi-transparent and brown, with metallic shiny, veins blackish-brown (except milk-white veins of basal part of membrane); basal part of first visible rostral segment, basal part of fore wing, veins of discal cell of corium, basal parts of veins of two cells of membrane, apical part of femur, basal part of tibiae, middle part of ventral side of abdomen, posterior margin of connexival segment milk-white; eyes yellowish-brown.

Structure: Median-sized. Body mostly clothed with yellowish-white depressed short setae; first antennal segment and basal half of second sparsely clothed with longer setae, dorsal-lateral side with several small tubers; apical half of second antennal segment, third and fourth segments with yellowish-brown short setae; legs sparsely with yellowish-brown setae of variable length (Figures 6a, b; and 7a–c). First antennal segment longest, apical part slightly bent; spines on head dorsally, spines on pronotum obviously stronger and longer; two spines on middle part of posterior pronotal lobe and two spines on middle-posterior part of anterior lobe vertical; post-lateral angle of abdominal connexivum angle-shaped produced post-laterally, apical part acute, and shape of connexivum changeable gradually in

different geographical populations, just as shown in Figure 2a–o. Pygophore elliptic, paramere clavate, apical part with thick setae (Figure 7d, e); phallosoma elliptic (Figure 7f–h); dorsal phallothecal sclerite sclerotized and flat, tongue-shaped (Figure 7c); number, shape and size of spine of endosoma changeable in different geographical population, nearly no spines or with very small pale spines (Figure 3a–e), or six spines on one side and five spines on the other side (Figure 3f, g), or four pairs of spines (Figures 3h; 7f–h), or five pairs of spines (Figure 3i–o).

Measurements [male (n= 139) / female (n= 90), in mm]: Body length 14.53±0.92 / 15.48±1.01, maximal width of abdomen 4.42±0.23 / 5.60±0.32. Head length 3.41±0.15 / 3.63±0.17; length of anteocular part 1.43±0.11 / 1.56±0.09; length of postocular part 1.47±0.08 / 1.50±0.11; length of synthlipsis 0.74±0.08 / 0.78±0.07; distance between ocelli 0.56±0.04 / 0.60±0.05; length of antennal segments I–IV= 6.42±0.34 / 6.31±0.34: 3.32±0.17 / 3.25±0.15: 2.08±0.17 / 1.95±0.12: 3.49±0.27 / 3.49±0.29; length of visible rostral segments I–III=1.22±0.06 / 1.27±0.05: 1.79±0.10 / 1.88±0.07: 0.56±0.06 / 0.59±0.06; length of pronotum 2.73±0.16 / 2.91±0.17; maximal width of pronotum 4.37±0.22 / 4.83±0.16; length of hemelytron 9.77±0.39 / 10.46±0.40.

Material examined. China, Anhui: 8 males (CAU, SE-AHFY, SE-AHFY1–5), Anhui, Chuzhou, Fengyang Mountain, 2008-VI-5; China, Chongqing: 1 male (CAU, SE-CQSMS1), Chongqing, Simian Mountain, 1200 m, 2017-IX-26; China, Fujian: 2 males, 5 females, 2009-VI-5, 1 male, 4 females, 2009-X-2, (CAU, SE-FJWY1–12), Fujian, Wuyishan, Tongmu, Cao Liangming leg.; 3 nymph, (CAU, SE-FJZZ1–3), Fujian, Zhangzhou, Nanjing, Huboliao Natural Reserve, 2018-VII-17, kept in CAU; 2 females (CAU, SE-FJJL1–2), Fujian, Jiangle, Longxi Moutain, 1991-X-10, Yang Jikun leg.; 2 males, 2013-IX-7, 1 female, 2014-VI-26, 1 male, 2014-VII-27 (CAU, SE-FJQZ1–4), Fujian, Quanzhou, Dehua, Shangyong, Houzhaicun; 1 male, 1 female, (CAU, SE-FJJY1–2), Fujian, Jianyang, Outou, 1974-X-26, Yang Jikun & Li Fasheng Leg.; China, Guangdong: 1 male, 1 female (CAU, SE-GDSG1–2), Guangdong, Shaoguan, Nanling, 2004-VII; 1 male, 1 female (CAU, SE-GDSG3–4), Guangdong, Shaoguan, Qujiang, Xiaokeng Natonal Forest Park, 2013-X-1, Zheng Chaowu leg.; 1 male, 1 female (CAU, SE-GDSG5–6), Guangdong, Shaoguan, Nanling, 2004-V-9; 1 male, 1 female (CAU, SE-GDHZ1–2), Guangdong, Huizhou, Longmen, Nankunshan, 2016-V-14–15; 3 males, 4 females (CAU, SE-GDLXH1–7), Guangdong, Liuxihe, 2004-VII-29, 2004-VIII-29; 1 male, 1 female (CAU, SE-GDCBL1–2), Guangdong, Chebaling, 2003-II-22; 1 male, 2016-V-01, 1 male, 2016-IV-29, 1 male, 1 female (CAU, SE-GDZC1–4), Guangdong, Zengcheng, Nankunshan, 2004-V-12; 15 males, 15 females (NNU, SE-GDQY1–30), Guangdong, Qingyuan, Yangshan,

2019-VIII-5, 200 m, Zhao Ping & Li Donghai leg.; 15 males, 15 females, 9 nymphs (NNU, SE-GDMZ1–39), Guangdong, Meizhou, Jiaoling, Longtai, 2019-IX-3, 355 m, Zhao Ping & Li Donghai leg.; China, Guangxi: 5 males, 4 females (NNU, SE-GXYL1–9), Guangxi, Yulin, Darong Mountain, 2018-VIII-19; 1 male, 1 female (CAU, SE-GXJX1–2), Guangxi, Jinxiu, 2006-X-15; 1 male, 1 female, 2011-VII-5, 2 males, 1 female, 2012-IV-8 (CAU, SE-GXJX3–7), Guangxi, Jinxiu, Dayao Mountain; 5 males, 9 females (CAU, SE-GXJX8–21), Guangxi, Dayao Mountain, 2015-IX-10; 1 male, 1 female (CAU, SE-GXLS1), Guangxi, Longsheng, Huaping, Hongtan, 2008-V-2; 1 female (CAU, SE-GXLS2), Guangxi, Longsheng, Huaping, 2018-V-2; 1 male (CAU, SE-GXLS3), Guangxi, Longsheng, Huaping, Hongtan, 1982-VI-26, Wang Xinli leg.; 1 male, 1 female, (CAU, SE-GXWM1–2), Guangxi, Wuming, Damingshan, 2011-V-25; 3 males, 5 females (CAU, SE-GXMES1–8), Guangxi, Maoershan, 2015-IX-22; 1 male, (CAU, SE-GXLG1), Guangxi, Lingui, 1996-VII-4, Shi Fuming leg.; China, Guizhou: 15 males, 20 females, 16 nymphs (KU, SE-GZLS2–5, SE-GZLS7–51), Guizhou, Leishan, Fangxiang, Pingxiang, 1050 m, 2018-VII-26, 2017-X-11, Zhao Ping & Li Donghai leg.; 15 males, 15 females (KU, SE-GZSB1–30), Guizhou, Shibing, Yuntaishan, 980 m, 2019-IV-6, 2018-VI-28, Zhao Ping & Li Donghai leg.; 10male, 10 females (KU, SE-GZML1–20), Guizhou, Maolan, 2013-X-12–15, Zhao Ping & Li Donghai leg.; 10 males, 10 females (KU, SE-GZLP1–20), Guizhou, Liping, Taipingshan, 2012-VIII-12, Zhao Ping leg.; 1 male (CAU, SE-GZXS1), Guizhou, Xishui, 2000-VI-2, Shi Fuming leg.; 1 male, 1 female (CAU, SE-GZFJS1–2), Guizhou, Tongren, Jiangkou, Fanjing Mountain, 2002-VI-3, 2012-X-1; 1 male (CAU, SE-GZLD1), Guizhou, Luodian, 1987-VIII-25, Li Fasheng & Yang Jikun leg.; 1 male, 1 female (KU, SE-GZDZ1–2), Guizhou, Danzhai, 2014-V-1, Zhao Ping leg.; 23 males, 26 females (NNU, SE-GZJP1–49), Guizhou, Jinping, Yandong, 834 m, 2018-VIII-29, Yuan Jilin leg.; China, Hainan: 2 females, (CAU, SE-HNBW1–2), Hainan, Bawangling, 2007-X-21; 1 male (CAU, SE-HN), Hainan, 2008-V-9; 1 female (CAU, SE-HNDLS1), Hainan, Diaoluoshan, 2007-X-27; China, Hunan: 2 males, 1 female (CAU, SE-HNFSS1–3), Hunan, Fushoushan National Forest Park, 2016-IX; 1 female (CAU, SE-HNSHS1), Hunan, Shunhuangshan National Forest Park, 2012-IV; 1 male (CAU, SE-HNTTS1), Hunan, Tiantaishan, 2004-X-4, Xu Zaifu leg.; 2 males (CAU, SE-HNCD1–2), Hunan, Changde; China, Jiangxi: 15 males, 34 females (KU, SE-JXNC1–49), Jiangxi, Nanchang, Meiling, 2018-VIII-14, Zhao Ping & Li Donghai Leg.; 15 males, 24 females (KU, SE-JXJG1–60), Jiangxi, Jinggangshan, 2018-VIII-16, Zhao Ping & Li Donghai Leg.; China, Sichuan: 1 female (CAU, SE-SCYA1), Sichuan, Yaan, 650 m; China, Taiwan: 6 females (CAU, SE-TW1, SE-TW3–7), Taiwan, Lijialindao, 2012-VI-3; 1 female (CAU, SE-TW2), Taiwan, Yilian, Fushan Natural

Reserve, 2013-VI-9; 2 male (CAU, SE-TWNT1–2), Taiwan, Nantou, 2010-XI-11; 1 male (CAU, SE-TWNT3), Taiwan, Nantou, Huisun Forest Farm, 2016-XI-5; 2 males, 2 females (CAU, SE-TWNT4–7), Taiwan, Lianhuachi Research Centre, 2012-VI-27; 2 males, 2 females (CAU, SE-TWPD1–4), Taiwan, Pingdong, Mudanxiang, 2012-VI-24U; China, Yunnan: 1 female (CAU, SE-YNPB1), Yunnan, Honghe, Pingbian, Dweishan, 2006-V-15, Wang Hesheng leg.; 1 female (CAU, SE-YNPB2), Yunnan, Pingbian, 2009-V-23; China, Zhejiang: 20 males, 20 females (KU, SE-ZJZC1–39), Zhejiang, Jiangshan, Ershibadu, Zhoucun. 2018-VIII-12, Zhao Ping & Li Donghai Leg.; 1 female (CAU, SE-ZJLS1), Zhejiang, Lishui; 1 female, Kualun, N.W. Fokien, 99.252, kept in BMNH; 1 female, N. China, 54.8., kept in BMNH. Vietnam: 3 males, 3 females (CAU, SE-VNM1–6), Vietnam, Phu Tho Province, Xuan Son National Park, 400m, 2004-X-14–20, Xuan Lam Truong Leg.

Distribution: China (Anhui, Chongqing, Fujian, Guangdong, Guangxi, Guizhou, Hainan, Hunan, Jiangxi, Sichuan, Taiwan, Zhejiang); Vietnam (Phu Tho).

Remarks: The shape of the connexivum and the number and the size of spines on the apical part of the endosoma, which could provide morphological evidence for species division of *Sclomina* (as described in the key above), are also obviously variable in different geographical populations of *Sclomina erinacea* (Figures 2a–o; and 3a–o). We tried to show the complicated differences with pictures in Figures 2a–o and 3a–o.

The lateral angle of the abdominal connexivum of *Sclomina erinacea* is angle-shaped and apical part is sharp (Figure 2a–o). However, the lateral angle of the connexivum in several groups from the southeast area of China (Figure 2a–e; JXNC, JXJG, ZJZC, FJQZ, GDQY samples; including GDMZ) is shorter than those in other populations, and the lateral angle is longer from the two island provinces of Taiwan and Hainan (TWNT and HNBW; Figure 2n, o).

Mostly, there are ten spines in the endosoma of the genitalia of *Sclomina erinacea* (Figure 3i–o, GZLS, GDSG, GXYL, GXJX, GZJP, TWNT, HNBW samples from Central China, and Taiwan and Hainan Islands). However, the endosoma in GZLP and GZHP is armed with eight spines (Figures 3h, 6e–g). The spines of the endosoma are pale and small in several populations from the southeast area of China (Figure 3a–e, JXNC, JXJG, ZJZC, FJQZ, and GDQY samples, including GDMZ). The endosoma of AHFY and CQSMS samples is armed with eleven spines (Figure 3f,g).

However, the genetic distance between individuals and populations of *Sclomina erinacea* was not significant compared to the five delimited species (Tables S3 and S4). The population change of this species deserves further analysis.

Symbiotic plant: *Rubus alceifolius*, *Rubus buergeri*, *Rubus lambertianus*, *Rubus rosifolius*, *Rubus setchuenensis*, *Rubus tsangorum*, *Rubus chiliadenus*, *Rubus tsangii*, *Rubus tephrodes*. Surrounding other symbiotic plants: *Boehmeria nivea*, *Hibiscus mutabilis*, *Macrothelypteris* sp., *Pilea sinofasciata*,

Polygonum senticosum, *Rhododendron rivulare*, *Sambucus chinensis*, *Solanum aculeatissimum*, *Woodwardia prolifera*, *Artemisia anomala*.

Sclomina guangxiensis **Ren, 2001**

(Figures 2p; 3p; 6d–f; 8)

Sclomina guangxiensis Ren, 2001: 1.

Diagnosis: This species *S. guangxiensis* is similar to *S. erinacea*, but in former the body color is dark reddish-brown, the posterior pronotal lobe is with black markings, and the post-lateral angles of the abdominal connexiva are long and narrow (vs. in *S. erinacea,* the body color is brown to yellowish brown, the posterior pronotal lobe is without black markings, and the post-lateral angles of the abdominal connexiva are shorter).

Redescription: Body Colouration: Body dark reddish-brown with black and yellow markings (Figures 6d, e; 8a–c). Apical and median parts of first antennal segment, basal half of second segment, apical half of third segment, fourth segment reddish-brown; two median annulations of first antennal segment, subapical annulation of second segment, median annulation of third segment pale yellowish-brown; apical part of second antennal segment, basal part of third segment black; longitudinal strips of head laterally, anterior half of head ventrally, anterior half of posterior pronotal lobe, ventral surface of fore femur, two longitudinal strips of mid and hind femur ventrally, two oblique stripes of tibiae, markings of coxae and tranchanters, propleural episternum, markings of meso- and meta-pleuron of thorax, longitudinal markings on lateral side of abdomen black; membrane of fore wing transparent and brown, vein dark brown (except basal vein yellowish to milk-white), middle part of corium black; basal half and posterior margin of connexivum yellowish-brown, posterior-lateral angle of each connexival segment dark-brown to black (except posterior margin pale); middle part of abdomen dull yellow, lateral side with black and yellow markings, sometimes scattered with reddish-brown. Structure: Body median-sized. Body covered with yellowish curved depressed short curved setae and sparsely clothed with long setae. First antennal segment longest, apical part bent. Spines on head dorsally, spines on pronotum obviously strong and long (Figures 6d, e; and 8a–c). Two long spines on middle part of posterior pronotal lobe bent backwards, two spines on middle-posterior part of anterior lobe bent forwards (Figure 8b); post-lateral angles of abdominal connexival segments long spine-shaped produced laterally (Figures 2p; and 8a–c). Pygophore elliptic, median pygophore process medianly concave (Figure 8d, e); paramere clavate, apical part with thick setae (Figure 8f); phallosoma elliptic, dorsal phallothecal sclerite sclerotized and flat, tongue-shaped (Figure 8g);

apical part of endosoma with two pair of large spines in middle part, and laterally with three spines on one side and four spines on the other side (Figures 3p; and 8g–j).

Measurements [male (n= 22) / female (n= 23), in mm]: Body length 14.63±0.17 / 15.7±0.35, maximal width of abdomen 4.77±0.13 / 5.28±0.60. Head length 3.49±0.16 / 3.74±0.14; length of anteocular part 1.48±0.12 / 1.60±0.09; length of postocular part 1.48±0.10 / 1.52±0.02; length of synthlipsis 0.72±0.05 / 0.74±0.05; distance between ocelli 0.57±0.04 / 0.61±0.05; length of antennal segments I–IV= 6.63±0.20 / 6.60±0.00: 3.21±0.15 / 3.21±0.14: 1.96±0.03 / 2.24±0.05: 3.30±0 / 3.74±0.09; length of visible rostral segments I–III= 1.21±0.00 / 1.21±0.05: 1.89±0.09 / 2.04±0.34: 0.57±0.04 / 0.55±0.00; length of pronotum 2.93±0.11 / 2.97±0.19; maximal width of pronotum 4.99±0.11 / 5.50±0.17; length of hemelytron 9.90±0.34 / 10.01±0.41.

Type material examined: Holotype: male, Guangxi, Daxin, 2800 m, 1998-III-19, Li Wenzhu leg., kept in NKU. Paratypes: 1 female, Guangxi, Ningming, Longrui National Natural Reserve, 1984-V-22, 200 m, Ren Shuzhi leg., kept in NKU; 1 female, Guangxi, Ningming, Longrui National Natural Reserve, 1984-V-19, Ren Shuzhi leg., kept in NKU.

Other material examined: China, Guangxi: 20 males, 20 females (CAU, SG-GXLZ1–40), Guangxi, Longzhou, Nonggang, 2017-VIII-22, 2017-XI-11, 2020-IV-30, Zhao Ping & Yang Mingyuan leg.; 1 male, 2 females (CAU, SG-GXLZ41–43), Guangxi, Longzhou, Nonggang, 2006-V-13, Huang Xia & Shi Zhongting leg.; 1 male, 1 female (CAU, SG-GXLZ44–45), Guangxi, Longzhou, Nonggang, 2006-X-4, Huang Xia leg.

Distribution: China (Guangxi).

Remark: In terms of the morphological evidences and the geographical distribution (Figures 1b; 2; and 3), we conjectured at first that *Sclomina guangxiensis* was an special case of the *Sclomina erinacea* cline and had serious doubt on taxonomic status as a valid species. Through the integrative taxonomy based on DNA barcoding and morphological evidences, the result showed that *Sclomina guangxiensis* is a valid species, and is far relative species and largest genetic distance with other members of *Sclomina* (Figures 1a,c; and 4).

Symbiotic plant: *Rubus amphidasys*, *Rubus lambertianus*.

Note: In addition, about the type specimens, Ren (2001) set up the species *Sclomina guangxiensis* Ren, 2001 based on one male and two female specimens and assigned the female specimen (Guangxi, Ningming, Longrui National Natural Reserve, 1984-V-22) as Holotype in the paper [6]. However, when we checked the type specimen kept in NKU, we found that the Holotype red label is under the male specimen (Guangxi, Daxin, 1998-III-19). It can be seen from her paper that most of the description and illustration of the species was based on this male specimen, so we chose this male specimen as Holotype herein. The

Holotype red label indicates that the type specimens are deposited in the Institute of Zoology, Chinese Academy of Sciences, but they are actually deposited in Nankai University (NKU).
